# Right upper lobectomy for lung cancer associated with a displaced anomalous bronchus: two case reports

**DOI:** 10.1186/s40792-024-01986-8

**Published:** 2024-08-15

**Authors:** Yoshihito Iijima, Takaki Mizoguchi, Masahito Ishikawa, Shun Iwai, Nozomu Motono, Hidetaka Uramoto

**Affiliations:** Department of Thoracic Surgery, Kanazawa Medical University, 1-1 Daigaku, Uchinada-Machi, Kahoku-Gun, Ishikawa, 920-0293 Japan

**Keywords:** Displaced right upper bronchus, Lung cancer, Right top pulmonary vein, Surgery

## Abstract

**Background:**

Bronchial bifurcation abnormalities are often discovered incidentally on chest computed tomography or bronchoscopy. As this condition is asymptomatic, it has little effect on the disease course of patients with lung cancer. However, this abnormality must be considered when performing lung resection.

**Case presentation:**

Patient 1 was a 73-year-old man with suspected simultaneous triple lung cancers [cT1c (3) N0M0, Stage IA3] in the right and left upper lobes. He was initially scheduled to undergo right upper lobectomy and systematic nodal dissection. Chest computed tomography revealed a displaced B^3^ that arose from the right middle lobe bronchus. V^1+2^ was transected first, followed by the superior truncus of the pulmonary artery, and B^1+2^, respectively. After the branches of V^3^ were ligated, B^3^ was identified smoothly. Finally, the incomplete interlobar fissure between the upper and middle lobes was separated using an auto-stapler. No vascular abnormalities were observed. Patient 2 was a 62-year-old woman with suspected lung cancer (cT1cN0M0, Stage IA3) in the right upper lobe, and was scheduled to undergo right upper lobectomy and lobe-specific nodal dissection. Chest computed tomography revealed a right top pulmonary vein and a displaced B^1^ that arose from the right main bronchus independently. Because V^1+3^ was resected simultaneously during upper and middle lobe resection during robot-assisted thoracic surgery, the procedure was cool-converted to video-assisted thoracic surgery. An independently A^1^ was observed, followed by A^2^_b_ and A^3^, which branched off as a common stem. A right top pulmonary vein was smoothly detected. Each blood vessel was transected using an auto-stapler. B^2+3^ was transected first using an auto-stapler, followed by B^1^.

**Conclusions:**

The displaced anomalous bronchus is often accompanied by pulmonary arterial or venous abnormalities and an incomplete interlobar fissure. A “hilum first, fissure last” technique is often useful. Preoperative evaluation and surgical planning are important.

## Background

Owing to the development and widespread application of imaging technology, such as three-dimensional (3D) computed tomography (CT), thoracic surgeons can obtain a precise understanding of the anatomical structures of the patient’s lungs and detect bronchial bifurcation abnormalities or branching anomalies of pulmonary vessels preoperatively [1, 2]. A displaced right upper bronchus (DRUB) is asymptomatic and has little effect on the progress of lung disease. However, these abnormalities must be considered when performing lung resection. Abnormal branching of the pulmonary vessels and bronchi is often encountered during pulmonary resections, and it is extremely important to discuss any abnormalities observed preoperatively on 3D-CT with the surgical team and formulate a proper surgical plan [2]. Herein, we report two cases of right upper lobectomy associated with DRUB.

## Case presentation

### Patient 1

**Fig. 1:**
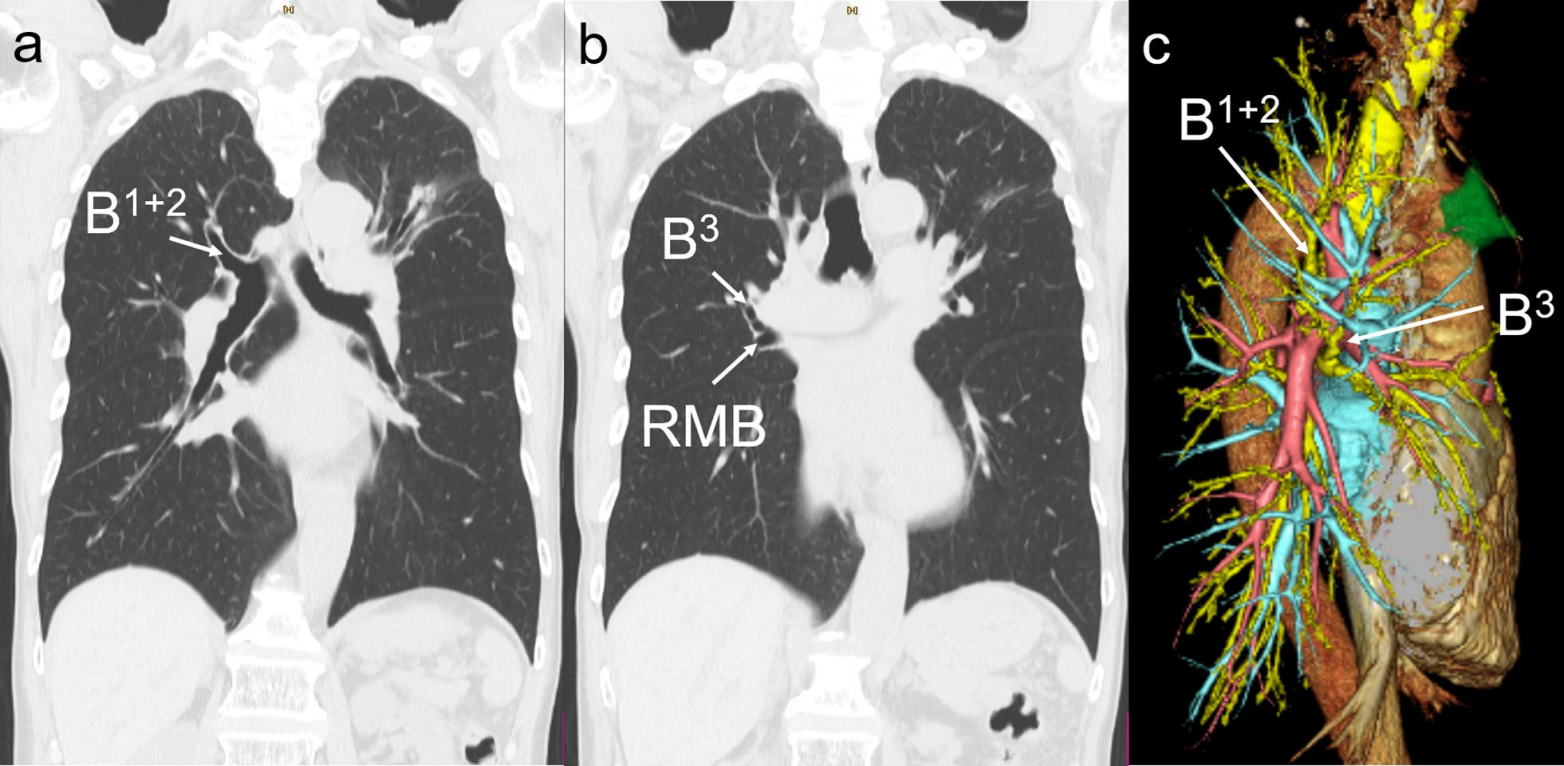
Preoperative findings of Patient 1. Chest computed tomography (CT) revealed: **a** an apicodorsal bronchus (B^1+2^) from the right main bronchus (RMB) and **b** a right ventral bronchus (B^3^) originating from the right middle lobe bronchus (MLB). **c** Three dimensional-CT broncho-angiography detected a displaced B^3^ originating from the right MLB and B^1+2^ from the RMB

**Fig. 2:**
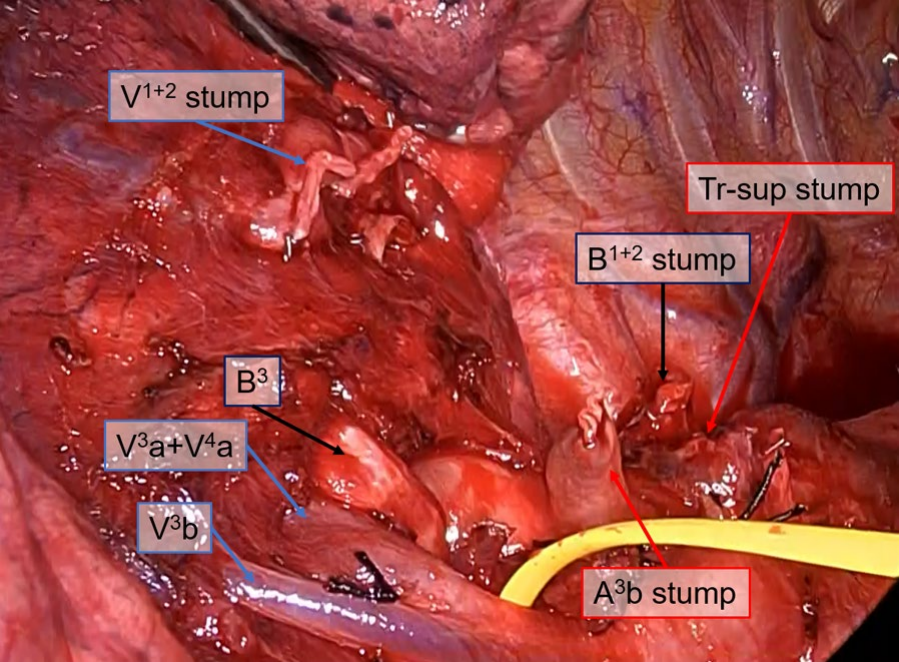
Intraoperative findings of Patient 1. After the common trunk of the apical vein and dorsal vein, the trunks superior to the pulmonary artery, and the apicodorsal bronchus were transected using an auto-stapler in that order. The ventral bronchus was detected smoothly

A 73-year-old male patient presented with an abnormal chest shadow during a routine health checkup. Adenocarcinoma was detected by sputum cytology. He was a former smoker, but otherwise had no notable medical history. Chest CT revealed a 25-mm solid nodule in the right ventral segment (S^3^), a 12-mm part-solid nodule in the apical segment (S^1^), and a 28-mm part-solid nodule in the left apicodorsal segment (S^1+2^). Therefore, simultaneous triple lung cancers were suspected. 3D-CT broncho-angiography (BAG) (Fig. 1) and virtual bronchoscopy (VB) detected an abnormality wherein the right ventral bronchus (B^3^) originated from the right middle lobe bronchus (MLB) and the apicodorsal bronchus (B^1+2^) from the right main bronchus (RMB). Transbronchial lung biopsy (TBLB) revealed adenocarcinoma in the nodule in the right S^3^. Right upper lobectomy (RUL) and systematic nodal dissection were performed using video-assisted thoracic surgery (VATS). Lobulation between the upper and middle lobes was incomplete. At the pulmonary hilum, the common trunk of the apical and dorsal veins, superior truncus of the pulmonary artery (PA), and B^1+2^ were transected using an auto-stapler. Next, the branches of the ventral veins were ligated. B^3^ was detected smoothly (Fig. 2) with light assistance from bronchoscopy. However, since the branch that was thought to be B^3^ and transected using an auto-stapler was actually B^3^_b_, B^3^ was transected in more central direction from the stump of B^3^_b_. Finally, near-infrared fluorescence imaging was performed by administering 5 mg of indocyanine green intravenously to visualize the demarcation line of the upper and middle lobes, which were separated using an auto-stapler. No complications were observed during surgery. The patient was diagnosed with simultaneous lung cancer, S^3^ adenocarcinoma (pT1cN0M0, Stage IA3) and S^1^ adenocarcinoma (pT1aN0M0, Stage IA1). The drainage tube was removed on postoperative day (POD) 2. He developed atrial fibrillation on POD 3 and underwent direct current defibrillation. He was discharged on POD 9. Left upper division segmentectomy and lobe-specific nodal dissection were performed on POD 35. The patient was diagnosed with a left upper lobe adenocarcinoma (pT1bN0M0, Stage IA2). The patient did not wish to receive adjuvant chemotherapy. Ten months after the initial lung surgery, the patient was undergoing outpatient follow-up.

### Patient 2

**Fig. 3:**
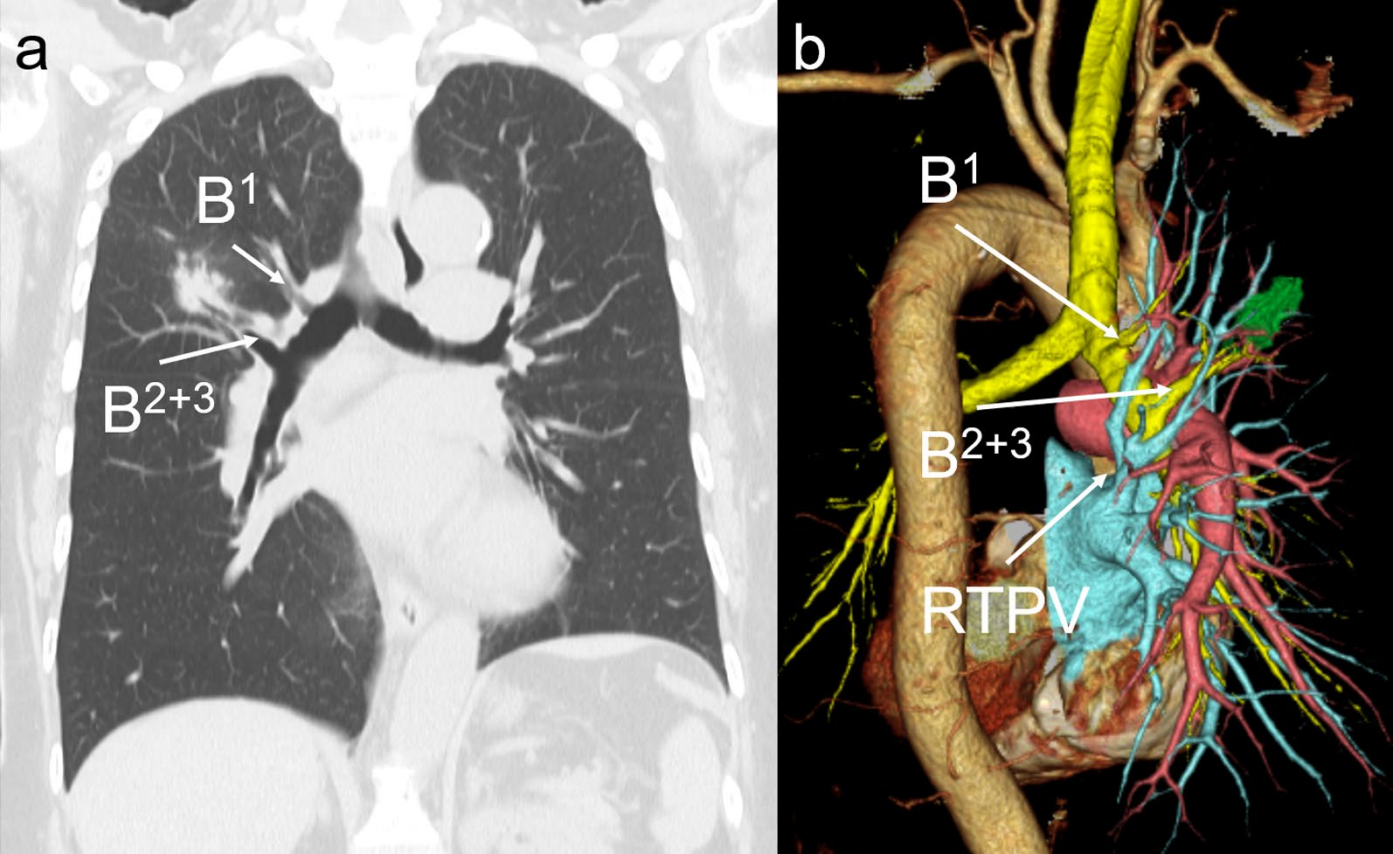
Preoperative findings of Patient 2. Chest computed tomography (CT) revealed: **a** a displaced right apical bronchus (B^1^) and common stem of the dorsal and ventral bronchus (B^2+3^) arising from the right main bronchus (RMB) independently, and **b** a right top pulmonary vein (RTPV)

**Fig. 4:**
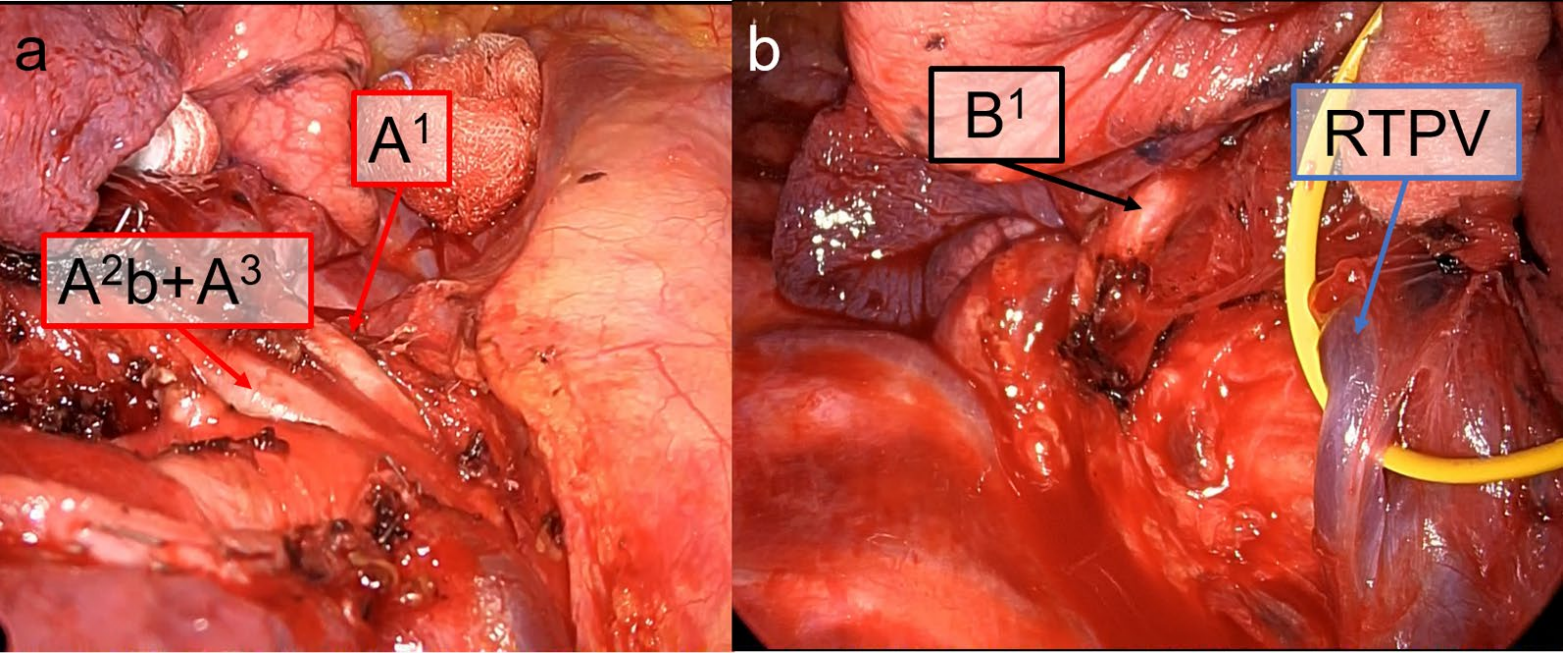
Intraoperative findings of Patient 2. **a** An apical artery (A^1^), with a common stem of the horizontal subsegmental artery (A^2^b) and ventral artery (A^3^), and dorsal subsegmental artery (A^2^a) branched separately. **b** At the dorsal side of the hilum, a right top pulmonary vein (RTPV) and displaced apical bronchus (B^1^) were detected

A 62-year-old woman presented with an abnormal chest shadow detected during a routine health checkup. She was a never smoker and had no notable medical history. CT revealed a 27-mm irregularly shaped nodule in the right dorsal segment (S^2^). 3D-CT BAG and VB imaging detected a displaced right apical bronchus (B^1^) and a common stem of the dorsal and ventral bronchus (B^2+3^) that arose from the RMB independently and a right top pulmonary vein (RTPV) (Fig. 3a, b). TBLB revealed that the nodule in the right S^2^ was adenocarcinoma. Right upper lobectomy and lobe-specific nodal dissection were performed using robot-assisted thoracic surgery (RATS). Lobulation between the upper and middle lobes was existent but poor. After exposing the main trunk of the PA at the inter-lobe, the upper and middle lobes were dissected using an auto-stapler. During interlobar resection, the apicoventral vein (V^1+3^) was simultaneously resected incidentally. Because the stump of V1 + 3 was involved in the staple line of the middle lobe, we decided to perform a cool conversion from RATS to VATS. The apical artery (A^1^) branched independently, followed by the horizontal subsegmental artery (A^2^_b_) and ventral artery (A^3^), which branched off as a common stem (Fig. 4a). Each branch was transected using an auto-stapler. While dissecting A^2^_b_ + A^3^, the interlobar lymph node (LN) (#11 s) around B^2+3^ was subjected to intraoperative frozen section biopsy, which tested negative. The RTPV was smoothly detected and transected using an auto-stapler (Fig. 4b). Thereafter, the upper and lower lobes were dissected using an auto-stapler. Finally, the B^2+3^ and B^1^ were transected by auto-stapler in this order. The patient was diagnosed with lung adenocarcinoma with segmental LN (#13) metastasis (pT1cN1M0, Stage IIB). The patient underwent adjuvant chemotherapy with cisplatin–docetaxel followed by osimertinib. Six months after surgery, the patient was undergoing follow-up without recurrence.

## Discussion

Bronchial bifurcation abnormalities are often discovered incidentally on chest CT or bronchoscopy. Previous studies reported that the incidence of displaced bronchi is 0.64–0.76% [3, 4]. Further, 75–84.8% of tracheobronchial anomalies are reportedly found in the right upper lobe [3, 4]. Ohta et al. found that only 0.0045% (59 of 13,222) of cases showed a displaced segmental bronchus; of these, 10 cases (16.9%) had B^3^ branching off from the MLB, as in Patient 1, and 8 cases (13.6%) had B^1^ branching off alone from the RMB, as in Patient 2 [3]. Yaginuma analyzed the chest CT scans of 6,072 patients and reported that a displaced bronchus was observed in 46 cases (0.76%). He further classified DRUB into 4 types: (i) the “right upper lobar type”, where the right upper bronchus arises from the lateral wall of the trachea; (ii) the “right B^1^ type”, where B^1^ arises from the lateral wall of the trachea or RMB; (iii) the “right B^2^ type”, where B^2^ arises from the bronchus intermedius; and (iv) the “right B^3^ type”, where B^3^ is absent from the right upper bronchus and arises from the right MLB [5].

Tables 1 and 2 summarize previously published case reports of lung resection for lung tumors associated with a displaced B^3^ from the RMB [6–10] and a displaced B^1^ from the right main bronchus in Japan [11–15], respectively. To date, displaced B^3^ and displaced B^1^ has been reported in seven cases each, including the current study. In the cases with displaced B^3^, no association was found with PV anomalies. An incomplete interlobar fissure (IF) between the upper and middle lobes was observed in all patients. Therefore, in right upper lobectomy, a “hilum first, fissure last” or “no-touch fissure” technique was often chosen [9], and the approach of transecting the incomplete IF first was not adopted [6–10]. It is reportedly important to transect the incomplete IF after preparing the bronchus in order to prevent postoperative air leakage and to confirm that the resected part does not include a displaced bronchus [10]. An incomplete IF was observed in Patient 1. Transecting B^1+2^ made it easier to unfold the upper lobe dorsoinferiorly, and B^3^ became easier to dissect. By first transecting the bronchus, it was easier to transect the incomplete IF. If it is difficult to identify the bifurcation of B^3^, it may be easier to dissect it by transecting the peripheral branches such as B^3^_b_ first. Among the cases with a displaced B^1^, 3 cases were complicated by the presence of the RTPV. Several studies did not provide a description of the lobulation between the upper and middle lobes, and the details were unknown; however, one case had incomplete lobulation and the lobulation in Patient 2 in this study was also poor. Previous studies have reported that bronchial abnormalities are accompanied by anomalies in PAs and PVs [4, 14–17]. Yaginuma et al. reported that patients with a RTPV have a high frequency of displaced bronchus in addition to incomplete fissure [4]. Katsumata et al. have reported cases in which A^1^, dorsal subsegmental artery (A^2^a), A^2^b, lateral subsegmental artery (A^3^a), and medial subsegmental artery (A^3^b) diverged individually [11]. In the present Patient 1, in addition to the superior truncus (A^1^ + recurrent A^2^), A^3^a and A^3^b branched separately. In present Patient 2, the A^1^, the common stem of A^2^b and A^3^, and A^2^a branched separately.

**Table 1:** Lung resection for lung tumor associated with a displaced right bronchus arose from middle lobe bronchus in Japan

Case	Age (yr)	Sex	PV anomaly	Minor fissure	Tumor location	Surgery	Reference
1	45	F	–	Incomplete	S1	RUL	6
2	69	F	–	Incomplete	S2	RUL	6
3	68	F	–	Incomplete	S1	RUL	7
4	68	F	–	Incomplete	S5	RML	8
5	90	F	–	Incomplete	S1	RUL	9
6	68	M	–	Incomplete	S5	S3 + RML	10
7	73	M	–	Incomplete	S3, S1	RUL	Case 1

*PV* pulmonary vein, *PA* pulmonary artery, *F* female, *M* male, *MLB* middle lobe bronchus, *RMB* right main bronchus, *ND* not described, *RUL* right upper lobectomy, *Seg* segmentectomy, *RUML* right upper and middle lobectomy

**Table 2:** Lung resection for lung tumor associated with an aberrant B1 from right main bronchus in Japan

Case	Age (yr)	Sex	PV anomaly	Minor fissure	Tumor location	Surgery	Author
1	69	M	–	Incomplete	S1	RUL	11
2	73	M	ND	ND	S1	RUL	12
3	81	M	–	ND	S2/3	RUL	13
4	69	F	+	ND	S2/S3	RUL	14
5	79	F	+	ND	S2/S3	RUL	15
6	79	F	ND	ND	S3	RUML	12
7	62	F	+	Poor	S3, S1	RUL	Case2

*PV* pulmonary vein, *PA* pulmonary artery, *F* female, *M* male, *ND* not described, *RUL* right upper lobectomy, *RUML* right upper and middle lobectomy

In our institution, between April 2016 and March 2024, 1,069 lung cancer surgeries were performed general anesthesia, including pleural biopsy and diagnostic thoracoscopy, of which 10 cases (0.9%) were found to have DRUB (Table 3). DRUB was classified according to Yaginuma's classification [5]. Chest CT was performed to assess the presence of the RTPV and mediastinal branches of the left PA. RTPV was found in 3 of 10 cases. The mediastinal branch of the left PA was also found in 3 of 10 cases and one case included a branch to the lower lobe. We compared the RTPV, left mediastinal lingular branch of the PA, and left mediastinal inferior lobar branch of PA in patients with or without a DRUB (Table 4). The Chi-square test was used for analysis, and *p* < 0.05 was considered significant. Patients with a DRUB were significantly more likely to have an RTPV (*p* < 0.001). Although it is not possible to draw a definitive conclusion due to the small sample size, it was suggested that patients with a DRUB may also have a left mediastinal inferior lobar branch of the PA (*p* < 0.001). When a DRUB is present, attention must be paid to the abnormal vascular course not only during surgery on the right side but also in surgery on the left side. When performing anatomical lung resections, such as lobectomy and segmentectomy in pulmonary surgery, it is extremely important to use 3D-CT to visualize branching abnormalities in the PAs, PVs, and bronchi [2]. Furthermore, it is important to share this information with the surgeons and anesthesiologists before surgery. This is because anesthesiologists must generally perform intraoperative management, such as bronchial toilets and selective segmental inflation [18], during lung resection.

**Table 3:** Lung resection for lung cancer associated with displaced right upper bronchus in our institute

No.	Age	Sex	Type of DRUB	RTPV	Other anomaly	Minor fissure	Laterality of cancer	Approach	Operation
1	80	M	RUB	–	–	Unknown	Lt	cVATS → thoracotomy	LUL
2	69	M	B2	+	Lt. A4 + 5	Poor	Rt	RATS	RUL
3	73*	M	B3	–	–	Incomplete	Rt	hVATS	RUL
4	68	F	B3	–	–	Incomplete	Rt	cVATS	RBS
5	49	F	B3	–	–	Incomplete	Rt	cVATS	RUML
6	82	M	B1	–	Lt. A4	Poor	Rt	cVATS	RUL
7	78	M	B1	–	–	Good	Rt	hVATS	RLL
8	67	M	B1	–	–	Unknown	Lt	hVATS	LPn
9	67	F	B1	+	Lt B4 + 5, Lt. A4 + 5 + 8 + 9	Unknown	Lt	hVATS	Wedge
10	62**	F	B1	+	–	Poor	Rt	RATS → cVATS	RUL

*DRUB* displaced right upper bronchus, *RTPV* right top pulmonary vein, *M* male, *F* female, *RUB* right upper bronchus, *Lt* left, *rt* right, *cVATS* complete video-assisted thoracic surgery, *hVATS* hybrid VATS, *RATS* robot-assisted thoracic surgery, *LUL* left upper lobectomy, *RUL* right upper lobectomy, *RBS* right basal segmentectomy, *RUML* right upper and middle lobectomy, *RLL* right lower lobectomy, *LPn* left pneumonectomy, Wedge wedge resection

^*^Patient 1,

^**^Patient 2

**Table 4:** Frequency of RTPV and left PA by presence or absence of displaced right upper bronchus

	DRUB		
	With *n* = 10	Without *n* = 1059	*p* value
RTPV n (%)	3 (30.0)	46 (4.3)	< 0.001
Lt. mediastinal lingular branch of PA n (%)	3 (30.0)	306 (28.9)	0.939
Lt. mediastinal inferior lobar branch of PA n (%)	1 (10.0)	1 (0.09)	< 0.001

*DRUB* displaced right upper bronchus, *RTPV* right top pulmonary vein, *PA* pulmonary artery

## Conclusions

We performed right upper lobectomies for lung cancer associated with a DRUB. When DRUB is present, attention must be paid to the incomplete IF and abnormal vascular course, especially RTPVs. A “hilum first, fissure last” technique is often useful. It is extremely important to discuss any abnormalities using 3D-CT before surgery within the team for proper surgical planning.

## Data Availability

All data generated or analyzed during this study are included in this published article.

## Abbreviations

3D: Three-dimensional
CT: Computed tomography
DRUB: Displaced right upper bronchus
S^3^: Ventral segment
S^1^: Apical segment
S^1+2^: Apicodorsal segment
B^3^: Ventral bronchus
BAG: Broncho-angiography
VB: Virtual bronchoscopy
B^1+2^: Apicodorsal bronchus
MLB: Middle lobe bronchus
RMB: Right main bronchus
TBLB: Transbronchial lung biopsy
RUL: Right upper lobectomy
VATS: Video-assisted thoracic surgery
PA: Pulmonary artery
POD: Postoperative day
B^1^: Apical bronchus
S^2^: Dorsal segment
B^2+3^: Common stem of dorsal and ventral bronchus
A^1^: Apical artery
A^2^b: Horizontal subsegmental artery
A^3^: Ventral artery
RTPV: Right top pulmonary vein
RATS: Robot assisted thoracic surgery
PV: Pulmonary vein
A^2^a: Dorsal subsegmental artery
A^3^a: Lateral subsegmental artery
A^3^b: Medial subsegmental artery
